# Infrared Thermography as a Non-Invasive Tool to Explore Differences in the Musculoskeletal System of Children with Hemophilia Compared to an Age-Matched Healthy Group

**DOI:** 10.3390/s18020518

**Published:** 2018-02-08

**Authors:** Axel Seuser, Karin Kurnik, Anne-Katrin Mahlein

**Affiliations:** 1 Practice for Rehabilitation, Prevention and Orthopedics, 53225 Bonn, Germany; axel.seuser@t-online.de; 2 Dr. von Hauner Children’s Hospital, University of Munich, 80337 Munich, Germany; karin.kurnik@med.uni-muenchen.de; 3 Institute of Sugar Beet Research, 37079 Göttingen, Germany

**Keywords:** hemophilia, infrared thermography, non-invasive, silent bleeds, hot-spot

## Abstract

Recurrent joint bleeds and silent bleeds are the most common clinical feature in patients with hemophilia. Every bleed causes an immediate inflammatory response and is the leading cause of chronic crippling arthropathy. With the help of infrared thermography we wanted to detect early differences between a group of clinical non-symptomatic children with hemophilia (CWH) with no history of clinically detected joint bleeds and a healthy age-matched group of children. This could help to discover early inflammation and help implement early treatment and preventative strategies. It could be demonstrated that infrared thermography is sensitive enough to detect more signs of early inflammatory response in the CWH than in healthy children. It seems to detect more side differences in temperature than clinical examination of silent symptoms detects tender points. Silent symptoms/tender points seem to be combined with early local inflammation. Using such a non-invasive and sensor-based early detection, prevention of overloading and bleeding might be achieved.

## 1. Introduction

Hemophilia is an inherited X chromosome-linked recessive disorder of coagulation that occurs due to a deficiency of the coagulation factors VIII or IX, and affects 1 in 10,000 of the male population in all ethnic groups [1]. Recurrent joint bleeds are the most common clinical feature in patients with hemophilia and the leading cause of chronic crippling arthropathy. Bleeding episodes can be prevented and arthropathy avoided thanks to replacement therapy with the missing coagulation factor given on regular long-term prophylaxis since childhood [2]. Nonetheless, despite effective prophylaxis and low bleeding rates, silent bleeds may still occur [2], triggering a vicious circle of biochemical and biomechanical reactions that cause irreversible joint damage on the long-term [3]. There is a lot of attention paid to the biochemical reactions, as blood-induced inflammation and damage of joint tissues [4]. Less attention is paid to other means of examination like silent symptoms associated to joints like tenderness of capsule or ligaments and functional disturbances during gait [3,5], which might be caused by bleedings. Every bleed causes an immediate inflammatory response [4]. Inflammation increases local blood flow and causes hyperthermia. Biochemical reactions are the first to occur immediately after bleeding. The early alterations in body biochemistry are very often not detected because the clinical scores usually adopted to examine joint status in patients with hemophilia are only able to detect late functional disturbances and inflammatory impairment like synovitis, joint effusion, reddening and increasing heat of the joint-surrounding skin. At this late stage rehabilitation is more difficult [6]. Therefore, early detection of inflammatory changes is crucial to implement very early conservative preventative approaches. 

Within this context, infrared thermography could be a simple tool to detect early inflammation of joints and joint associated structures. Infrared thermography detects the emitted radiation of objects in different bands, roughly from 9000–14,000 nm. The temperature information is shown in false color images, known as thermograms. Every image pixel correlates with a temperature value, visualized in intensity maps. Thermography has been already successfully applied for the detection of plant diseases [7], in veterinary medicine for early detection of inflamed joints in horses [8] and in human medicine for early discovery of breast cancer [9]. It measures inflammation activity in different settings as prevention, rehabilitation and sports [10]. Painful muscle insertion of the extensor muscles at the elbow is associated with hot areas on a thermogram [11]. Thermal imaging can detect persistent tendon insertion problems of the elbow region in a similar way as isotope bone scanning [12]. Hot spots at the elbow have also been described as having a high association with a low threshold for pain on pressure [13]. The experience with thermography in patients with hemophilia is still limited, although it can be safely used in this setting because it is non-invasive [14,15,16]. The aim of this study was to demonstrate the use of thermography in identifying local rise in temperature in male children with hemophilia who never reported a clinical detected joint bleed and compare it to a group of healthy age matched boys. The second aim was to compare the two groups with their clinical results using the clinical examination score HJHS and looking for silent symptoms/tender points. 

## 2. Materials and Methods

### 2.1. Thermographic Image Acquisition and Clinical Investigation

Ten male children with severe hemophilia with a mean age of 10.3 years with no reported joint bleeds and no clinically detectable symptoms and 12 healthy boys aged 11.8 years on average were examined by infrared thermography. The body mass index showed no significant differences with mean values of 17.1 and 19.3 for hemophilic and healthy children, respectively (student’s *t*-test *p* = 0.127; Supplementary Material, Figure S1). Thermal images were obtained using a VARIOSCAN 3201 ST (Jenoptic Laser, Jena, Germany) Sterling-cooled IR scanning camera with a spectral sensitivity ranging from 8 μm to 12 μm and a geometric resolution of 1.5 mrad (240 × 360 pixels focal plane array and a 30° × 20° field of view lens with a minimum focus distance of approximately 20 cm). Thermal resolution was 0.03 K, and accuracy of absolute temperature measurement was <±2 K. Emissivity was set to 0.98.

**Figure 1:**
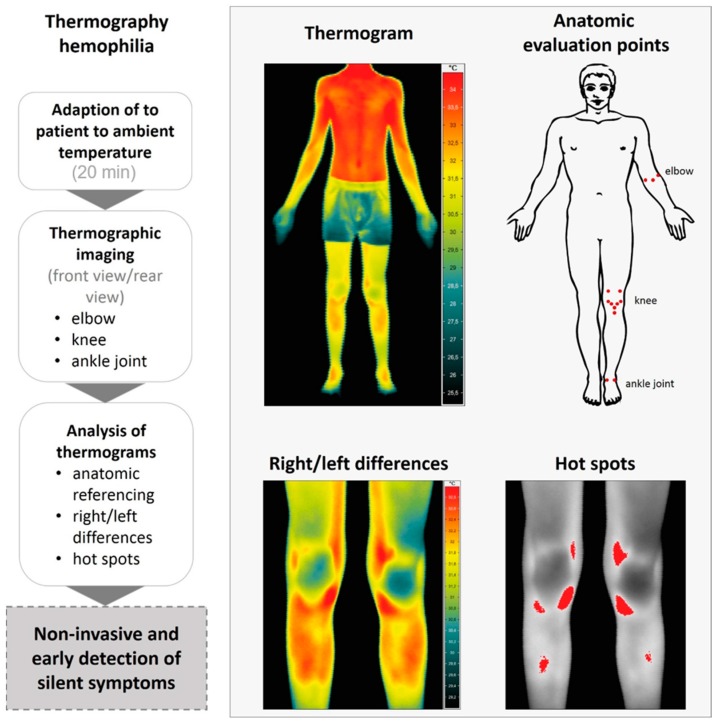
Workflow of thermographic image assessment and analysis of children with hemophilia (**left**) and example images for a termographic overview and close up images (**right**). After adaption period of 20 min to ambient temperature, thermograms of the individual anatomic points were assessed in front and rear view (**right**). According to anatomic reference points, right/left differences were calculated and correlated to clinical examination. Afterwards a confusion matrix was used to assess the accuracy, specificity and sensitivity of thermography compared to clinical examination.

For standardization of thermographic measurements, the Glamorgan protocol for recording and evaluation of thermal images of the human body was adapted to the specific measurement situation (looking at right/left differences) [17]. The measuring process and examples of obtained data are visualized in Figure 1. Room temperature was 21.9 °C in average and humidity 62.3%. The distance between the camera and the patients was around 90 cm for close up imaging, constantly. In addition a full body image was measured from a distance of 250 cm. A time period of at least 20 min before thermographic investigation was assured for adaption to room temperature for each patient. At first elbows, knees, and ankle joints were examined using thermograms with 86.400 Pixel. Afterwards the full body view was captured. Thermal images were taken from the front and from the rear view. Digital RGB images (Olympus PEN, E-P3, Olympus, Tokio, Japan) were taken in same position for anatomical comparison. In pre -tests with different patients we took side views as well of elbows, knees and ankles. As all the structures were visible enough to calculate right/left side differences in the front and back view from a defined region of interest, we decided to leave the Glamorgan protocol to reduce examination time for the children.

The boys were routinely asked not to do any sports or workouts three days before measurement. The examinations were routinely done between 10:00 and 12:00 on Saturdays (no school) after breakfast before daily activities.

After infrared thermography, both groups were examined with the commonly used clinical examination score (Hemophilia Joint Health Score (HJHS) which looks for joint swelling, joint pain, motion restriction, joint crepitus and atrophy of muscles) [6] and searched for silent symptoms/tender points (symptoms not felt by the patient but detectable by clinical examination such as tenderness provoked by palpation of the joint capsule, ligaments and tendons) [18]. 

In the front view the following anatomic points were evaluated: (i) the ulnar and radial epicondylus and elbow pitch; (ii) lateral and medial recessus, medial and lateral collateral ligament, medial and lateral Hoffa, apex patellae and tibial tuberosity of the knee; (iii) medial and lateral capsule on the ankle joint (Figure 1). Evaluation in the rear view included: (i) the radial and ulnar epicondylus, medial and lateral capsule of the elbow; (ii) medial collateral and lateral collateral ligament, dorsal medial and lateral capsule for knee; (iii) achilles tendon, tibialis posterior and peroneal tendon around the ankle joint (Figure 1).

Thermographic images were analyzed and temperature values of the joints and hot spots were extracted using the software IRBIS control. 

Temperature values of anatomical relevant regions were manually extracted by polygons. These polygons had the same size at both corresponding regions. Temperature difference are defined by more than 0.5 °C between the same anatomical regions on both sides [19]. Asymmetric rise of temperature in the same anatomical regions of the left and right ankles, knees and elbows were considered in detail. For individual visualization hot-spots are shown using a threshold value. These values were not used for further analysis. An example data set of thermographic investigations of a hemophilic child can be found in the Supplementary Material, Figure S2.

The study was carried out in accordance with good clinical practice and the Declaration of Helsinki. All the used data were a part of a retrospective study case series derived from routine follow-up visits. Data were anonymous in concordance with general data protection rules. Thermography is established as a normal procedure in the German medical fee schedule. The healthy control group was gathered by including male siblings of the patients that usually attend the practice appointments of their brothers upon the request of their parents. Informed consent from the parents was given for their data to be collected and used for comparison.

### 2.2. Statistical Analysis

The distribution of all continuous variables was tested by the Shapiro-Wilk test and equal variance was tested on by the Brown-Forsythe test accordingly. Continuous variables were compared by the Student’s *t*-test, in cases, equal variance was not given, the Mann-Whitney Rank Sum Test was applied. A *p* value < 0.05 was considered as statistically significant. Analyses were performed by using IBM SPSS Statistics software (release 21, IBM Corp., Armonk, New York, NY, USA). A confusion matrix was used to assess the precision (positive predictive value PPV), sensitivity, specificity (true negative value) accuracy and negative predictive value (NPV) comparing tender points and side differences in infrared thermography. For all sites of tenderness, the rates of true and false positive or negative cases was calculated in patients and controls. For each joint, the rates was summarised and then the rates for the investigated samples were determined. Finally, sensitivity and specificity of increased temperature for tenderness was calculated. 

## 3. Results

**Figure 2:**
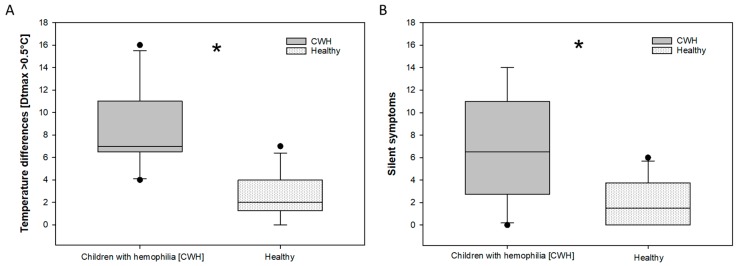
Thermographic side differences (>0.5 °C) (**A**) and corresponding silent symptoms/tender points (**B**) found in the groups of hemophilic and healthy children. Significant differences in temperature differences (**A**) were assessed by a student’s *t*-test. Significant differences in the amount of silent symptoms (**B**) were analyzed using the Mann-Whitney Rank Sum Test. Asterisks marks indicate significant differences with a *p* = <0.05.

Regarding the HJHS, no significant differences between children with severe hemophilia and the group of healthy children were observed. The clinical scores of the HJHS were zero (0) in each patient and all healthy boys. The examination for silent symptoms/tender points showed a significant difference (Figure 2B): 2.1 silent symptoms per child in the healthy group, versus 6.9 silent symptoms in the children with severe hemophilia were observed. There is a significant difference between children with severe hemophilia with no clinical symptoms and healthy children concerning thermography.

**Figure 3:**
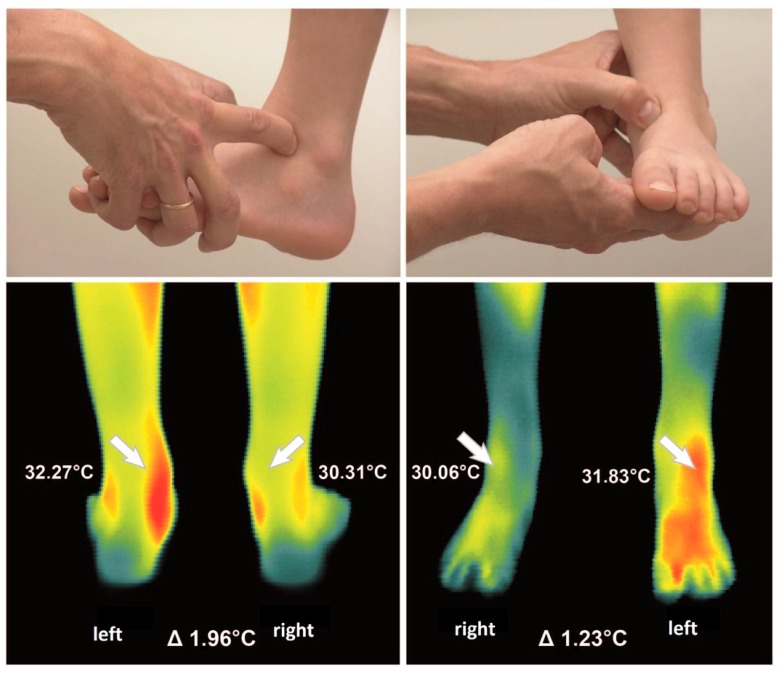
Example of a clinical examination of the antero-lateral and antero-medial capsule of the ankle joint (**upper part**). The thermographic examination of right/left differences of the ankle joint in front and rear view (**below**) showed marked right/left differences of 1.96 °C in the rear view on the tibialis posterior tendon and of 1.23 °C in the front view on the antero-lateral capsule.

Thermographic side differences (>0.5 °C) were found in both groups. Thermographic right/left differences are exemplarily shown in Figure 2 and Figure 3 In the healthy group the average was 2.6 (2.06 stdv.) per child. In the hemophilic group 8.3 (3.498 stdv.) relevant thermographic side differences per child have been found. More temperature differences than silent symptoms were observed in both groups.

**Figure 4:**
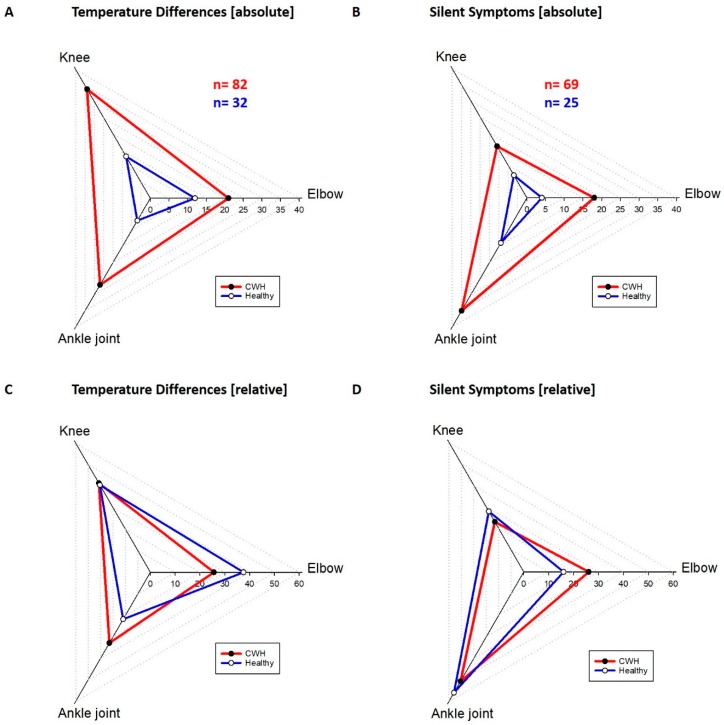
Absolute distribution of temperature differences (**A**) and silent symptoms (**B**) in children with hemophilia and healthy children. The sum of temperature differences was in the hemophilic group 82 and in the healthy group 32. 69 silent symptoms appeared in hemophilic children and 25 in healthy children. The relative appearance of temperature differences (**C**) and silent symptoms (**D**) among the different joints for healthy children and children with hemophilia.

The distribution of silent symptoms and thermographic right/left differences were different for the joints but similar in both groups (Figure 4). The elbows manifested 26.1% of all silent symptoms in the hemophilic group and 16% of all silent symptoms in the healthy group. The thermography result for the elbows was positive in 25.6% in the hemophilic group and 37.5% in the healthy group. From all silent symptoms found in CWH the knees of the hemophilic group carried 23.2% and in the healthy group 28%. The thermography of the knees showed 41.5% of the temperature differences >0.5 °C in the hemophilic and 40.6% in the healthy group. The ankles carried most of the silent symptoms with 50.7% for the hemophilic group and 56% for the healthy group. The thermography was positive on the ankle joints in 32.9% of the hemophilic group and 21.9% of the healthy group. The absolute number of temperature differences was 82 in the hemophilic group and much higher compared to the healthy group with 32 (Figure 4A). A clear difference was found also in the number of investigated silent symptoms, 69 silent symptoms were found in all CWH and 25 in all healthy children.

Additional results arise from the calculation of precision (positive predictive value), sensitivity, specificity (true negative value) accuracy and negative predictive value (NPV) comparing tender points and side differences in infrared thermography. After calculating the results for all investigated structures the results were pooled for higher numbers. All results for CWH and healthy children were added jointwise. 

The elbow showed the following results: Precision (positive predictive value) = 0.59, sensitivity = 0.94, specificity = 0.81, accuracy = 0.83, and NPV = 0.99. Knee joint: Precision (positive predictive value) = 0.16, sensitivity = 0.8, specificity = 0.68, accuracy = 0.70 and NPV = 0.985. Ankle joint: Precision (positive predictive value) = 0.51, sensitivity = 0.96, specificity = 0.71, accuracy = 0.77 and NPV = 0.98. All patients and all joints combined: Precision (positive predictive value) = 0.33, sensitivity = 0.84, specificity = 0.73, accuracy = 0.74 and NPV = 0.96.

## 4. Discussion

The latest study on arthrorrhagia in patients with haemophilia described infrared thermography as a possible tool to detect joint bleeds. It was possible to discriminate between a mild bleeding group and a moderate to severe bleeding group. [16] It is very important to have a sensitive tool for diagnosing acute bleeding episodes. It is equally important to know if infrared thermography can be used as a diagnostic tool at the onset of hemarthropathy as well.

The main focus of this study was to find a way to discover signs of inflammation in children with hemophilia in a very early, pre-clinical stage. Therefore we looked for a group of CWH with no clinical symptoms and no history of clinical joint bleeds and compared them with an age-matched group of healthy children. Early detection of inflammation could help making treatment more effective and prevent crippling deformation of the joints. Warmth is a cardinal feature of inflammation and may be objectively measured by the use of infrared thermography. There are studies that show a significant correlation between thermographic findings and disease activity in rheumatoid arthritis [15]. 

The normal degree of thermal asymmetry between opposite sides of the body is very small. Reported differences for the forehead were 0.18 ± 0.18 °C, for the leg 0.27 ± 0.2 °C, and for the foot 0.38 ± 0.31 °C. The values were measured over a period of 5 years and were obtained from 40 matched regions of the body surface of 90 asymptomatic normal individuals. These values could be used as a standard and the degree of asymmetry is a quantifiable indicator of dysfunction [20].

Significant asymmetry of more than 0.5 °C is not physiologic [19]. Subclinical problems can be diagnosed before clinical relevance can be seen [21]. Studies on horse tendons showed thermographic hot spots two weeks before swelling, pain and laming occurred [8]. In sports medicine thermography is used to discover pre-seasonal hot spots, for example in the tibial tuberosity of juvenile alpine skiers [22]. In 35 women and 52 male athletes thermography was performed. In seven of them the medium temperature difference on the tibial tuberosity was 1.4 °C recognized as hot spots. The mean temperature difference in the not affected athletes was 0.3 °C. Only four of the seven thermographically spotted patients reported symptoms like pain. The others were asymptomatic. The physical examination showed, that the hyperthermia correlated with a tender point on the tibial tuberosity.

The results indicate that thermography is a sensitive clinical diagnostic tool. It seems to detect more side differences in temperature than clinical examination of silent symptoms detects tender points in children with severe hemophilia and healthy children. Therefore it can be used to verify early local inflammation. The literature shows that a hot spot is correlated with an increase of local blood flow. An increase of local blood flow is used to identify inflammation or acute (silent) bleedings. Within this study, it was demonstrated, that there is a significant difference between silent clinical symptoms and thermographic right/left differences >0.5 °C in healthy children and a group of children with severe hemophilia without manifest clinical symptoms and no clinical bleeding history. Using only the HJHS score [6] for clinical examination none of the children would have shown any pathologic findings and could not be differentiated from the healthy group. Infrared thermography and examination for tender points can be used to describe early symptoms and then initiate preventative treatment. Especially for hemophiliacs it is necessary to start a preventative treatment before manifest hemarthropathy. The results in literature implicate, that silent bleeds occur [2,5]. It is known that even small amount of blood could lead to structural changes in early adulthood. Not only for the knee but especially for the ankle joints. There is an increasing number of patients with first clinical symptoms on their ankle joints in the mid-twenties [23].

The distribution of silent symptoms and of thermographic side differences in the different joints is rather similar between the two groups. Whereas the elbow and the knee together represent more or less half of the silent symptoms, the ankle joint in both groups was responsible for the other half of the silent symptoms. This could be due to a larger number of proprioceptive organs in the ankle joint versus the knee and the elbow joint. It could be one explanation why the ankle joint is showing earlier clinical signs of hemarthropathy than the other joints [23]. The knee joints show the highest number of thermographic right/left differences in both groups and less silent symptoms. This could indicate that it is not as sensitive to physical examination as the other joints. The knee might react to overloading with an increased blood flow before inflammation increases the sensitivity of the structures. The joint surrounding capsule and ligaments do carry proprioceptive sensors that are necessary for optimal joint function [24]. Even slight inflammation can disturb this sensible task and lead to malfunction, consecutively to repetitive stress, more overloading and finally structural damage [25]. 

Infrared thermography already proofed to be an early predictor of increased blood flow [8,15,22] and act in a similar way as isotope bone scanning [12] That allows to correlate the presence of tender points with the difference in temperature which reveals inflammation [13]. Overall more thermographic side differences were measured than tender points. This decreased the precision (positive predicting value) and indicates a medium to low reproducibility of the used test. But it indicates as well that infrared thermography is able to detect right/left differences in blood flow earlier than silent symptoms/tender points occur, particularly in the knee joints. Better values were found for specificity (true negative value) and for negative predictive value (NPV). This indicates that the joints and the neighboring structures can be predicted healthy with a good reliability, if no tender point and no thermographic side differences were found. A good sensitivity (low false negative results) and accuracy (describing accurately both true positive and true negative results) could recommend infrared thermography and clinical examination of tender points as reliable diagnostic tools for early interpretation of inflammatory response of the subcutaneous joint structures as capsule, ligaments and tendons. 

Physical therapy offers enough opportunity to treat preventatively and according to the findings. In further studies the success of the treatment could be evaluated by the same two diagnostic tools and can provide a quality control feedback. After the first paper on hemophilia and thermography from 1975 by Forbes et al. [26] this is a new—and because of the improved accuracy of thermographic cameras—a more successful attempt to implement it into clinical routine. The short comings of this paper are the small number of retrospectively evaluated patients and control group. We have to judge the interpretation of the data as preliminary. This is especially true for the calculated validity, reproducibility, reliability and diagnostic ability of the used tests. More studies and higher numbers are needed to get more reliable information.

## 5. Conclusions

Infrared thermography is a useful tool for early detection of inflammatory response differences of the joints in CWH and a healthy group of age-matched children. It detects more side differences in temperature than clinical examination of silent symptoms detects tender points in children with severe hemophilia. Early detection of inflammation based on non-invasive sensor measurements in addition with clinical examination of tender points could provide prevention of overloading and bleeding.

## Supplementary Materials

The following are available online at http://www.mdpi.com/1424-8220/18/2/518/s1, Figure S1: Body mass index of healthy children and children with hemophilia; Table S2: Example data set from region of interest extracted from thermographic images; Table S3: Results from the calculation of accuracy, sensitivity and specificity.
